# Challenges and potential solutions to the evaluation, monitoring, and regulation of surgical innovations

**DOI:** 10.1186/s12893-019-0586-5

**Published:** 2019-08-27

**Authors:** Derek J. Roberts, David A. Zygun, Chad G. Ball, Andrew W. Kirkpatrick, Peter D. Faris, Matthew T. James, Kelly J. Mrklas, Brenda D. Hemmelgarn, Braden Manns, Henry T. Stelfox

**Affiliations:** 1 Division of Vascular and Endovascular Surgery, Department of Surgery, University of Ottawa, The Ottawa Hospital, Civic Campus, Room A280, 1053 Carling Avenue, Ottawa, Ontario K1Y 4E9 Canada; 2 Division of Critical Care Medicine, Department of Medicine, University of Alberta, Edmonton, Alberta Canada; 3 Department of Surgery, University of Calgary, Calgary, Alberta Canada; 4 Department of Oncology, University of Calgary, Calgary, Alberta Canada; 5 Alberta Health Sciences Research – Research Analytics, University of Calgary and the Foothills Medical Centre, Calgary, Alberta Canada; 6 Department of Medicine, University of Calgary, Calgary, Alberta Canada; 7 Department of Community Health Sciences, University of Calgary, Calgary, Alberta Canada; 8 Strategic Clinical Networks, System Programs, and Innovation, Alberta Health Services, Calgary, Alberta Canada; 9 Department of Critical Care Medicine, University of Calgary, Calgary, Alberta Canada

**Keywords:** Debate, Evidence-based surgery, Pharmaceutical innovation, Surgical innovation, Surgical research

## Abstract

**Background:**

As it may be argued that many surgical interventions provide obvious patient benefits, formal, staged assessment of the efficacy and safety of surgical procedures has historically been and remains uncommon. The majority of innovative surgical procedures have therefore often been developed based on anatomical and pathophysiological principles in an attempt to better manage clinical problems.

**Main Body:**

In this manuscript, we sought to review and contrast the models for pharmaceutical and surgical innovation in North America, including their stages of development and methods of evaluation, monitoring, and regulation. We also aimed to review the present structure of academic surgery, the role of methodological experts and funding in conducting surgical research, and the current system of regulation of innovative surgical procedures. Finally, we highlight the influence that evidence and surgical history, education, training, and culture have on elective and emergency surgical decision-making. The above discussion is used to support the argument that the model used for assessment of innovative pharmaceuticals cannot be applied to that for evaluating surgical innovations. It is also used to support our position that although the evaluation and monitoring of innovative surgical procedures requires a rigorous, fit-for-purpose, and formal system of assessment to protect patient safety and prevent unexpected adverse health outcomes, it will only succeed if it is supported and championed by surgical practice leaders and respects surgical history, education, training, and culture.

**Conclusion:**

We conclude the above debate by providing a recommended approach to the evaluation, monitoring, and regulation of surgical innovations, which we hope may be used as a guide for all stakeholders involved in interpreting and/or conducting future surgical research.

## Background

As it may be argued that many surgical interventions provide obvious patient benefits, formal, staged assessment of the efficacy and safety of surgical procedures has historically been and remains uncommon [1]. The majority of innovative surgical procedures, defined as “a new or modified surgical procedure that differs from currently accepted local practice, the outcomes of which have not been described, and which may entail risk to the patient” have therefore often been developed based on anatomical and pathophysiological principles in an attempt to better manage clinical problems [1]. For example, damage control laparotomy, or abbreviated laparotomy followed by temporary abdominal closure and planned re-operation after a period of intensive care unit (ICU) resuscitation, was developed in response to the recognition that severely injured trauma patients frequently died from a “viscious cycle” of hypothermia, acidosis, and coagulopathy when a prolonged, definitive laparotomy was completed in a single stage [2]. Although the patient, system, and societal effects of the integration of damage control laparotomy into surgical practice were significant, its adoption was based on very little evidence comparing it to definitive trauma laparotomy [3, 4].

In this manuscript, we sought to review and contrast the models for pharmaceutical and surgical innovation in North America, including their stages of development and methods of evaluation, monitoring, and regulation. We also aimed to review the present structure of academic surgery, the role of methodological experts and funding in conducting surgical research, and the current system of regulation of innovative surgical procedures. Finally, we sought to highlight the influence that evidence and surgical history, education, training, and culture have on elective and emergency surgical decision-making. The above discussion is used to support the argument that the model used for assessment of innovative pharmaceuticals cannot be applied to that for evaluating surgical innovations. It is also used to support our position that although the evaluation and monitoring of innovative surgical procedures requires a rigorous, fit-for-purpose, and formal system of assessment to protect patient safety and prevent unexpected adverse health outcomes, it will only succeed if it is supported and championed by surgical practice leaders and respects surgical history, education, training, and culture. We conclude the above debate by providing a recommended approach to the evaluation, monitoring, and regulation of surgical innovations, which we hope may be used as a guide for all stakeholders involved in interpreting and/or conducting future surgical research.

## Main text

### Approval and monitoring of innovative pharmaceuticals in North America and the central role of the industry-funded, placebo-controlled randomized controlled trial (RCT)

Assessment of innovative pharmaceuticals within the United States and Canada occurs within a highly structured model regulated by the U.S. Food and Drug Administration (FDA) and Health Canada, respectively [5]. The development, approval, and monitoring of drugs within this model occurs within phases typically beginning with animal studies characterizing their pharmacology and safety across a dosage range (Phase 0) [1, 5]. The manufacturer uses this information to create an Investigational New Drug (IND) application, which is submitted to the FDA or Health Canada and must be approved before human studies begin [5]. After approval of an IND for use in humans, three phases of clinical safety and efficacy studies are conducted; at least two of which typically involve placebo-controlled RCTs [1, 5]. As these placebo-controlled RCTs are often expensive, funding is commonly supplied by the manufacturer [5]. Importantly, during this process, the innovation (characterized by Phases 0–3) and adoption and monitoring phase (characterized by Phase 4) of drug development, relate to events separated in time [1].

Phase I studies are conducted within a small cohort of healthy volunteers and assess the safety, tolerability, accepted dosage range, and pharmacokinetics of the IND after one or more doses [1, 5]. Phase II studies are often RCTs and the first studies conducted within patients with the target health condition [1, 5]. These trials administer the drug over its anticipated target dosage range and commonly assess feasibility and/or its influence on a biomarker or biomarkers [1, 5]. Phase III studies enroll hundreds to thousands of patients with the target condition into RCTs and are designed to assess the efficacy (if the trial is designed as an explanatory trial) or less often effectiveness (if the trial is designed as a pragmatic trial) of the medication on a series of pre-defined efficacy and safety endpoints [1, 5]. These trials are used by the FDA and Health Canada for market approval of the drug for one or more indications [1, 5]. Phase IV studies are post-marketing studies conducted to monitor for rare adverse effects or to assess the usefulness of the agent among other patient populations or when administered in different dosage forms [1, 5].

### The process of development for innovative surgical procedures

In contrast to the highly structured and regulated model of pharmaceutical innovation, the development of innovative surgical procedures remains largely unregulated, unstructured, and variable [1, 6, 7]. Despite this, development of many surgical innovations commonly proceeds unconsciously or unknowingly through a series of developmental and evaluative stages described in detail by the Balliol Collaboration in 2009 [1]. This group described four distinct stages (0–4) of surgical innovation, which need not occur sequentially, within the context of a conceptual framework known as the IDEAL (Innovation, Development, Evaluation, And Long-term implementation and monitoring) model (see Table 1 for a comparison of the models of pharmaceutical and surgical innovation) [1].

**Table 1:** Phases/Stages and Methods of Pharmaceutical and Surgical Innovation^1, 5, 7^

Phase/Stage	Pharmaceutical Innovation	Surgical Innovation
0	▪ Preclinical animal studies used to characterize the pharmacology of the agent as well as its safety across a dosage range ▪ Used to support creation of an IND application, which is submitted to the FDA or Health Canada	▪ Initial prehuman (simulator or animal) work and development, which may be reported as preclinical studies
1	▪ Phase I RCTs conducted within a small group of health volunteers to assess the safety, tolerability, accepted dosage range, and pharmacokinetics of the IND after one or more doses	▪ Technical skills development and/or acquistion and proof of both concept and safety, which may be described in structured case reports
2	▪ Phase II RCTs that administer the drug over its anticipated target dosage range to patients with the target health condition to assess feasibility and/or the infleunce of the drug on a biomarker or biomarkers	
2a		▪ Although the technical details of the surgical procedure have not been completey refined, a few surgical practice leaders have adopted the technique and are using it on a small group of patients outside the index hospital or center where it was originally developed ▪ May be described in prospective development studies
2b		▪ Many of the technical details have been nearly perfected, and the surgeons who adopted the procedure in stage 2a start to broaden patient accrual and procedural indications ▪ May be described in cohort studies, diagnostic performance studies, and RCTs
3	▪ Phase III RCTs designed to assess the efficacy or effectiveness of the medication in patients with the target health condition on a series of pre-defined efficacy and safety endpoints	▪ The innovative procedure is now becoming part of many surgeon’s practices, and only a select few will not have adopted it ▪ May be evaluated in RCTs or other studies where clinical equipoise exists
4	▪ Post-marketing studies conducted to monitor for rare adverse effects or to assess the usefulness of the agent among other patient populations or when administered in different dosage forms	▪ Long-term monitoring studies whose aim is to assess for unexpected rare outcomes and restrict or expand indications for the procedure or clarify additional important technical details

Where FDA indicates U.S. Food and Drug Administration; *IND* Investigational New Drug, and *RCT* Randomized controlled trial

In the IDEAL model, stage 0 refers to the initial pre-human (simulator or animal) work and development, whereas stage 1 is the first time the innovative procedure is performed on a small group of highly selected patients [1]. The focus of stage 1 is largely technical skills development and/or acquisition and proof of both concept and safety [1, 7]. Frequently, a technique that may have been developed or used in one surgical specialty is adapted for use in another specialty. Stage 2 is subdivided into stages 2a and 2b [1]. In stage 2a, although the methods or technical details of the surgical procedure have not yet been completely refined, a few surgical practice leaders have adopted the technique and are using it on a small group of patients outside the index hospital or center where it was developed [1, 7]. Stage 2b begins when many of the technical details have been nearly perfected, and the surgeons who adopted the procedure in stage 2a broaden patient accrual and procedural indications [1]. In stage 3, the innovative procedure is now becoming part of many surgeon’s practices, and only a select few will not have adopted it [1]. It has also now been described for use among different groups of patients or for different indications than originally proposed [1]. Finally, stage 4 is similar to phase 4 of pharmaceutical innovation, which consists of long-term monitoring studies whose aim is to assess for unexpected rare outcomes and to restrict or expand indications for the procedure or clarify important technical details [1].

### The contrasting pattern of RCT use for the evaluation of surgical innovation

In contrast to the central role of the RCT in evaluating the efficacy and safety of innovative pharmaceuticals, few RCTs are currently available that directly compare surgical procedures to placebo/sham surgery or even to alternative surgical or interventional procedures [1, 6, 8]. One systematic review reported that only 8.5% of all publications reported among three surgical journals (*Diseases of the Colon & Rectum*, *Surgery*, and the *British Journal of Surgery*) in the year 2000 were RCTs and that this percentage increased to only 10% in 2010 [9]. Further, many “surgical” RCTs involve evaluations of perioperative medications or resuscitation/monitoring techniques rather than comparisons of surgical procedures [1, 7]. Studies of surgical procedures conducted among humans are therefore often uncontrolled and frequently consist of retrospective, single-arm cohort studies, a study design with at least a moderate risk of bias [6].

### Challenges in using RCTs for the evaluation and regulation of surgical innovations

#### Differences in the process of innovation for surgical procedures versus pharmaceuticals

In contrast to pharmaceutical innovation, the development of surgical procedures continues to occur well after its adoption into practice, often making it difficult to decide when to move from a developmental stage (i.e., IDEAL stage 1 and 2) to one of formal validation [6]. If this transition occurs too early, the attempted constraints of procedure definition and standardization within an RCT may hinder innovation [6]. However, if done too late the state of clinical equipoise (i.e. uncertainty regarding the benefits or risks that may result from use of the innovative versus conventional or already existing procedure) may be lost among practicing surgeons, precluding random allocation of patients to the innovative procedure versus another intervention [6]. Further, in contrast to an IND approved for use in humans, the development of the involved surgical methods and skills that constitute an innovative surgical intervention often continue throughout the innovation of the procedure [6]. Any less than encouraging findings during its early evaluation may therefore reflect the surgical learning curve rather than its efficacy and safety [6]. Thus, in contrast to the requirement for demonstration of evidence of efficacy and safety during the early innovation of a novel pharmaceutical, the role of the RCT is likely minimized in many cases of surgical innovation until at least stage 3 or the early phase of adoption for the procedure (i.e., when its technical details have been worked out and the learning curve has largely passed) [1].

#### Methodological and technical challenges for use of RCTs for surgical evaluation

In addition to the difficulty in deciding when to evaluate an innovative surgical procedure, several special methodological or technical problems may also partly explain the deficiency of RCTs comparing alternate surgical interventions [6, 10]. As patients frequently demand the latest technology advertised through media or other sources, and surgeons are sometimes keen to adopt novel techniques, surgical trials may be limited by patient or surgeon preference for interventions with a suspected or perceived improvement in benefit-to-risk profile [6, 11]. For example, although endovascular abdominal aortic aneurysm repair requires life-long endograft surveillance and may have less favorable long-term outcomes than open repair [12], even younger patients with abdominal aortic aneurysms who are fit for major operative intervention may decline traditional open repair as they have heard about the purported benefits of minimally invasive repair from their social circle, the media, or on the internet. Moreover, as a result of an imbalance of experience favoring an established surgical intervention, comparison of an innovative procedure with an established standard surgical intervention is also often criticized [6]. Finally, as the surgical methods employed during each procedure vary (sometimes considerably) between surgeons, the exact definition of the procedure as well as any attempts to limit excessive inter-procedural variation may be difficult, which may make interpretation, duplication, and/or generalization of results difficult [10].

The innate variability in anatomy and presenting pathology seen among patients may also may make surgical RCTs more difficult to conduct, interpret, and generalize [13]. Further, when there are only small differences between the surgical method used in the experimental versus control procedure, a large study sample may be required to demonstrate superiority, making the RCT an inefficient design [6]. Similarly, while several component changes to a surgical procedure over time may ultimately produce improved clinical outcomes, an RCT that evaluates only one of these component changes may not observe improved efficacy or safety [10]. Thus, patient outcomes could potentially be adversely influenced in some cases if randomized evidence was required to “prove” that one or another component change in the technique of an operation produced improved patient outcomes [10]. Further, although randomization should ideally be done as close as possible to the time of surgical intervention to prevent cross-over between the treatment and comparison groups, this may sometimes be challenging or impossible in practice [6].

Finally, although a surgical procedure is principally conducted by the surgeon and therefore influenced by their decision-making and technical skill, the outcome of the procedure is also dependent on other operating room team members (surgical residents/fellows, anesthesiologists, nurses, and technicians) as well as the quality of perioperative management [6]. As an example, in addition to the varied surgical technique involved in damage control laparotomy (e.g., the indications used for the procedure, the operative interventions performed, and potentially the time spent in the operating theatre), other preoperative (e.g., the exact indication used for the procedure, use of damage control resuscitation techniques, and/or administration of tranexamic acid) and postoperative interventions (e.g., intensive care and/or angiographic embolization) may differ between treatment groups and contribute to the outcomes observed between the treatment groups [6]. Thus, the quality of reporting of surgical RCTs is contingent upon describing perioperative care interventions in detail. Despite this, a cross-sectional survey of 120 two-arm parallel RCTs assessing surgical interventions indexed in PubMed in 2013 suggested that less than 40% of these trials provided data on anesthetic management or postoperative care [14]. Improvements in the quality of surgical intervention reporting are therefore needed.

#### Unique challenges for the conduct of RCTs in trauma and emergency surgery

Several additional difficulties may limit the design and conduct of RCTs of emergency surgical procedures, including those for trauma. As evidence is often lacking in many of these areas, and for other reasons that will be discussed below, surgical dogma and the opinions of surgical practice leaders likely significantly influence surgeon preference for one procedure over another in emergency situations [6]. These preferences may preclude randomization due to surgeons’ lack of equipoise [6]. Moreover, as the surgical intervention must be performed urgently, and study patients often present outside of normal daylight working hours (frequently precluding availability of key research staff), the conduct, management, and logistics of an RCT design can be demanding for surgical investigators [10]. Further, although some investigators may argue that a waiver of consent should be granted where informed or surrogate consent may not be feasible nor practical to obtain, and where clinical equipoise exists, such waivers of consent may sometimes be difficult to obtain from institutional research ethics boards (IREBs), making trial execution difficult if not sometimes impossible [6, 10]. Finally, as emergent surgical patients who meet the exact inclusion/exclusion criteria of the RCT may only present intermittently, recruitment of the required sample size of patients may take years or be overly costly and thus sometimes unfeasible [10].

#### Lack of external validity among published surgical RCTs

In addition to the methodological and technical challenges to conducting RCTs in surgery, the application of the evidence afforded by these studies is often limited by an actual or at least *perceived* lack of external validity [15–17]. Although RCTs are widely cited as providing the most robust evidence on the efficacy of an intervention under ideal circumstances, they sometimes provide less evidence on the effectiveness of the procedure or its performance in routine practice [16–18]. Trials may exclude elderly patients and/or those with multiple co-morbidities or specific prior interventions (including surgery), or they may appear to have been conducted within a highly controlled environment [18]. For example, trials comparing laparoscopic versus open hernia repairs frequently exclude patients with obesity, cirrhosis, and concurrent infection as well as those who received previous hernia repair or were requiring emergency operation, all of which are commonly encountered in practice [19]. Thus, some surgeons may argue that the results of these trials are not generalizable to or helpful for the patients they encounter in their practices [15–17].

#### Evidence-based surgery and surgical history, education, training, and culture

Aside from the challenges outlined above for conducting RCTs in surgery in a manner similar to pharmaceuticals, several characteristics of surgical history, education, training, and culture may limit the perceived importance or use of evidence (randomized or other) by practicing surgeons [6, 13, 20]. Very few of the surgical procedures in use today were validated by RCTs or other controlled trials demonstrating their efficacy/effectiveness and/or safety [10]. Thus, for procedures widely adopted into practice soon after their development, it may be impossible for researchers to compare their efficacy and safety against another procedure viewed to be inferior given the lack of equipoise [10]. This problem is likely greatest when two procedures proposed to be compared in an RCT are perceived to have substantially different benefit-to-harm profiles (e.g., minimally invasive versus traditional open procedures) [6] or when surgeons perceive that not giving the intervention will produce a poor outcome (e.g., definitive instead of damage control laparotomy for patients with a juxtahepatic venous injury or persistent intraoperative acidosis) [21, 22].

The traditional “master-student” or apprenticeship model of surgical training has also been described as being much less conducive to evidence-based practice than the present model of medical training [6]. According to Ergina and colleagues, the staff or attending surgeon is described in this model as being viewed as the “master” who already possesses the required surgical knowledge, which can only be gained by the resident apprentice through “observation and emulation” [6]. Thus, residents learn by observing and memorizing methods by which each of their attending surgeons (or other surgical practice leaders, through surgical textbooks, conferences, and other tertiary literature sources) conducts an operation.

Although the above model works well for learning many different crafts, it may influence the conduct or translation of research not supported by attending surgeons or surgical opinion leaders [6]. These issues are likely further compounded within the context of trauma and emergency surgery given the paucity of high-level evidence, and the difficulty in studying emergent surgical conditions or learning how to manage them without an adequate period of apprenticeship with a master surgeon. Thus, despite attempts at knowledge translation strategies, implementation or deimplementation of practices in surgery (e.g., inappropriate use of surgical drains or various postoperative feeding practices) remains a slow and ineffective process without the support or oversight of appropriate master surgeons or surgical practice leaders for the reasons outlined above [6]. This also implies that evidence-informed practice changes may be more effectively achieved in surgery than in medicine through the engagement of recognized surgical practice leaders in programs of research.

Actual or perceived barriers or impediments from single surgical practice leaders or entire surgical practice groups may also restrict the adoption of best evidence into surgical practice even when evidence suggests that patient outcomes are improved with an alternate approach [13]. This phenomenon is widely known, and captured by the well known surgical phrase “you can’t do that here because we don’t do that here” [13]. Further, as the practice of surgery involves constant, close interprofessional interactions, surgical decisions may be substantially swayed (consciously or unconsciously) by the opinions of colleagues or fear of a repeat poor outcome because of a bad experience had when managing another patient [13]. For example, although there is no randomized evidence to support the routine use of percutaneous surgical drains after routine or emergent laparoscopic cholecystectomy [23], some surgeons may still choose to leave drains in patients as they have been influenced by previous anecdotes of patients suffering one or more poor outcomes as a result of a missed postoperative bile leak.

### Academic surgery, methodological expertise, and research funding

As surgery is a craft improved through practice and experience, academic surgeons must devote more time to clinical service than their academic physician colleagues to maintain competence, especially when their chosen specialty is more technically demanding. This limits their available time to conduct research and write grant funding applications [6]. Moreover, although the vast majority of surgeons are familiar with the principles and methods of epidemiology and biostatistics, a lack of formal training in evidence-based medicine among many surgeons may contribute to an inability to find (i.e., because of a lack of training on how to conduct effective electronic database searches) and/or interpret (owing to a lack of critical appraisal skills) the best-available evidence. It likely also leads to a lack of desire to conduct RCTs or other more complicated epidemiological study designs [10]. Further, while research funding is required for many academic surgeons in order to design and conduct RCTs and other studies of surgical procedures, some evidence exists to suggest that surgeons are less likely to apply for non-industry funding, and also less likely to be successful when they do [6, 10]. Finally, in contrast to the situation with pharmaceuticals, unless an innovative surgical procedure involves use of a device, industry funding is often unavailable to surgeons.

### Present systems for regulation of innovative surgical procedures

As surgical innovation exists in a “grey zone” between interventions intended to provide therapeutic benefit to patients and research studies designed to “test an hypothesis [sic], permit conclusions to be drawn, and thereby to develop and contribute to generalizable knowledge”, it is frequently challenging to define an innovative surgical procedure as an intervention or research [11]. Thus, unless the surgeons involved with the innovation decide to present it in the form of research (in which case they would have to be submit their work to an IREB), no formal regulatory framework exists for evaluating innovative surgical procedures [1, 11].

In part to address this problem, a formal document was issued by the National Commission for the Protection of Human Subjects of Biomedical and Behavioral Research in 1979 known as the Belmont Report [11]. Although this report stated that when surgical procedures “depart in a significant way from standard or accepted practice the innovation does not, in and of itself, constitute research” that would require a formal research study, it did state that “radically new procedures” should mandate such evaluations [11]. However, as the terms “significant” and “radically new” remain ill-defined, the process of surgical innovation in North America remains largely self-regulated by the surgical profession unless it involves use of a medication or medical device, under which circumstances it would have to follow the regulations afforded by the FDA or Health Canada [1, 11].

### The IDEAL recommendations for evaluation and monitoring of surgical innovation

As a practical system for the development and evaluation of surgical procedures that does not unnecessarily hinder surgical innovation is required, the Balliol Collaboration created recommendations in 2009 that coincide with their proposed model of surgical innovation described above [7]. The Collaboration recognized that it may be impossible to change the process of surgical innovation, and instead decided to adapt their development and evaluation methods to the existing process [7].

In the IDEAL recommendations, the Balliol Collaboration recommended that when an innovative procedure is first being used among humans (stage 1), the surgeons involved inform the hospital of the intention to perform the novel procedure [7]. They also recommended that surgeons report all innovative procedures, including both failures and successes, ideally in an online register that is freely available and accessible to all surgeons [7]. Prior to the beginning of stage 2a (where a few surgical leaders have begun to use the technique among a small group of patients), the IDEAL recommendations also suggest that protocols for prospective development studies are designed and registered *before* patient recruitment commences [7]. These protocols should describe patient indications and contraindications, operative methods, and a priori outcome measures [7]. As technical modifications and a learning curve are expected during stage 2a, all changes in surgical method should be recorded and consecutive outcomes should be reported for all cases without omissions [7]. Finally, the IDEAL recommendations suggest reporting “selection criteria and proportion of eligible cases selected; a clear description of the procedure and each modification, with timing; and relevant outcomes, with recognized standard definitions of important categories, such as specific complications” [7]. In stage 2b, where the surgeons who adopted the procedure in stage 2a start to increase patient accrual and procedural indications, prospective research databases should be established such that data can be collected on all patients receiving the innovative procedure [7]. These databases should contain well-characterized technical, clinical, and patient-reported outcomes, and could be used to conduct prospective uncontrolled studies and design RCTs [7]. They should also be used to describe the patient population presenting for treatment, and how many were treated by the innovative versus conventional or other procedures [7]. During stage 3, where the procedure is becoming part of many surgeons’ practices, it should be evaluated for effectiveness and safety against a current standard, ideally with an RCT [7]. The recommended method for monitoring during stage 4 (long-term monitoring) is use of a registry that captures only well-defined relevant information and key outcomes [7]. After adjusting for case-mix or known potential confounding factors between the groups, analyses may be done to investigate outcome variations between subgroups among large numbers of patients accumulated over time [7].

## Conclusion

### A recommended approach to the evaluation, monitoring, and regulation of innovative surgical procedures

As the development of innovative surgical procedures continues to occur long after its initial use among humans, the approval and monitoring of innovative surgical procedures cannot occur in sequential stages as in the model of pharmaceutical regulation and monitoring [1, 7]. Moreover, as “unrealistically demanding standards could hinder surgical innovation,” the requirement for large RCTs demonstrating efficacy/effectiveness and safety prior to their use in clinical practice may have a detrimental influence on surgical progress [7]. Despite this, the evaluation and subsequent monitoring of innovative surgical procedures requires a formal system to prevent recurrent mistakes and protect patients [7]. Table 2 outlines some potential solutions to the challenges of evaluating, monitoring, and regulating surgical innovations, including some of those suggested in the IDEAL recommendations [7].

**Table 2:** Potential Solutions to the Challenges of Properly Evaluating, Monitoring, and Regulating Innovative Surgical Procedures

Research Methods and Funding Challenges	
▪ Increased funding opportunities (alone and within research teams) and expert methodological support for surgical researchers ▪ Increased support by Departments of Surgery and their members to recruit, support, and retain academic surgeons ▪ Increased use of RCT (including pragmatic, adaptive, tracker, expertise-based, or cluster), experimental or quasi-experimental (parallel group, non-randomized, controlled interrupted time-series studies, or stepped wedge designs by surgery or site), and comparative effectiveness studies for evaluating surgical innovations (guided by the above methodological experts) (where RCTs are either unethical or impractical) ▪ Increased use of the IDEAL recommendations for evaluating surgical innovations by surgeons, institutions, scientific journals, and other stakeholders ▪ Editors of surgical journals and professional surgical societies should mandate that studies of surgical innovations be reported according to the EQUATOR guidelines and that EQUATOR checklists are uploaded with studies when submitted for peer-review ▪ Increased conduct and reporting of economic analyses of surgical interventions to determine their cost-effectiveness (ideally these studies would be “piggy-backed” to RCTs comparing an innovative to a conventional surgical procedure)	
Surgical History, Education, Training, and Culture	
▪ Integration of formal education on evidence-based medicine knowledge and skills into surgical residency training programs ▪ Increased CME and surgical journal series on evidence-based surgery topics for staff surgeons and surgical trainees ▪ Increasing the number of surgeon and non-surgeon researchers in Departments of Surgery with formal training in research methodology ▪ Increased support by surgical opinion leaders on a shift towards a culture of surgical practice that is based on evidence *and* apprenticeship ▪ Use of knowledge translation interventions that embrace that surgical practice changes and the use of evidence in surgery may occur more effectively when championed or supported by surgical practice leaders ▪ Research to better understand the methods by which surgeons make decisions and decide to implement or de-implement evidence-informed practices into or out of surgery	

Where CME indicates continuing medical education; *EQUATOR* Enhancing the QUAlity and Transparency Of health Research, *IDEAL* Innovation, Development, Evaluation, and Long-term implementation and monitoring, and *RCT* Randomized controlled trial

Ideally, surgical innovations should be first reported in case reports and then case series and subsequently evaluated in cohort studies followed by RCTs and economic analyses. Although RCTs are recommended for evaluating surgical innovations by IDEAL, the conduct of these trials may be limited by methodological and technical challenges as well as issues related to external validity and cost. As such, if RCTs are to be viewed as an ideal component in the process of innovation for many surgical procedures, methods should be created that allow for increased funding opportunities (either alone or within research teams) and expert methodological support for surgical investigators. As the conduct of surgery in health care is resource intensive (especially when postoperative ICU care is required), and may contribute to bodily disfigurement and mortality, it represents a significant cost item and surgeons must advocate for funding for academic surgeons and Departments of Surgery to properly and ethically study the effects of surgical innovations on patients. Moreover, before any surgical intervention is adopted its cost-effectiveness should be assessed in economic analyses [24]. Ideally, a cost-effectiveness analysis should be “piggy-backed” to an RCT comparing an innovative to a conventional surgical procedure.

While use of pragmatic, adaptive, or cluster (i.e., investigators may instead randomize clusters of patients to an intervention performed by an experienced individual surgeon or center rather than allocating the intervention at a patient-level) RCTs may increase the external validity of trials [18], conduct of these studies may not be feasible or even required in some situations. In these cases, other types of experimental or quasi-experimental studies, including parallel group non-randomized studies, controlled interrupted time-series studies, stepped wedge designs by surgery or site (i.e., randomizing a group of surgeons to do the procedure in an innovative way and another group of surgeons to do the procedure in the traditional or comparative way), tracker trials, or expertise-based RCTs may be used [7]. These designs would likely often require that surgeons had equipoise regarding the superiority of the two procedures and that they are appropriately trained to perform the procedure in both the innovative and traditional way. Observational studies using various comparative effectiveness research methodologies that aim to provide causal inference may also be helpful when RCTs are not feasible for ethical or pragmatic reasons [7, 18]. These methodologies include propensity scores, marginal structural models, and instrumental variables analysis [18]. While these studies cannot prove causation, they may be used to highlight the need for RCTs or support increased or decreased use of an intervention until RCT evidence becomes available.

However, if evidence-based surgery is to play a larger role in surgical decision-making and the evaluation and regulation of surgical procedures, some authors have suggested that changes in surgical education must first occur [25]. First, surgeons must be taught how to effectively search for and critically appraise existing evidence. Although changes have already begun to occur in North America with the integration of formal education on evidence-based surgery into surgical residency training programs, these teachings are likely insufficient [24, 25]. They also ignore many of the non-evidence-based barriers to surgical decision-making discussed herein [25, 26]. Surgical journals may assist surgeons and surgical trainees in learning evidence-based medicine knowledge and skills by publishing specialized series dedicated to surgical audiences. For example, over the past two decades, the *Canadian Journal of Surgery* published the Users’ Guides to the Surgical Literature, which contains articles guiding surgeons on how to find and evaluate surgical evidence [27–30].

However, only with the support of senior surgical leadership may it be possible to facilitate a shift in the culture of surgical practice from one that is based largely on apprenticeship to one that is also based on evidence *and apprenticeship* [25]. In surgery, where the opinions of surgical practice leaders are so highly regarded, their involvement in individual programs of surgical research may greatly improve the translation of the work produced and improve both the implementation and de-implementation of surgical practices. Finally, despite arguments that the model should be changed completely from one based on apprenticeship to one based on evidence [25], we would argue that apprenticeship-type learning will always be at least partly required for practices that are difficult to study (e.g., minor variations in intraoperative or postoperative care practices) and high-risk surgical situations where evidence is unavailable and decisions must be made under conditions of uncertainty and significant time-constraints [20]. In these situations, surgeons learn from their mentors to synthesize existing information and draw on past experiences and knowledge to make high quality decisions.

In addition to support from surgical leaders, several additional actions may facilitate improvements in the evaluation and reporting of surgical innovations or procedures (Table 2) [7]. These include promotion of the IDEAL standards for study reporting and design by journal Editors as well as calls for IDEAL study designs by journals [7]. Editors may also assist with creating registries of surgical protocols and reports [7], and provide opportunities to publish these works in surgical journals. Although “domain-specific” (i.e., surgery-specific) funding is rare, research funding agencies could also develop specific funding sources for appropriately designed studies of surgical innovation, and provide support for design of surgical databases, registries, and reporting media [7]. Regulating bodies could also accept IDEAL study designs as the appropriate method of evaluation for surgical innovation, provide rapid and flexible ethical reviews of innovative surgical procedures during the early stages of innovation, and link provisional approval to appropriate registration of evaluation of all innovative procedures [7]. Further, professional surgical societies could ensure that all guidelines follow the IDEAL recommendations, and even require members to report innovative procedures in registers in order to maintain specialty recognition [7]. Finally, in addition to recommending that the “level” of evidence afforded be explicitly reported in articles published in their affiliated journals, Editors of surgical journals and surgical societies should mandate that studies of surgical innovations be reported according to the Enhancing the QUAlity and Transparency Of health Research (EQUATOR) guidelines [31]. They may also mandate that the relevant EQUATOR checklist be uploaded as supplemental digital content when studies are submitted for peer-review (available at http://www.equator-network.org/). Only with all or many of the very significant changes outlined above, would such a system of formal evaluation and monitoring of surgical innovation be widely adopted into practice.

## Data Availability

Not applicable.

## Abbreviations

EQUATOR: Enhancing the QUAlity and Transparency Of health Research
FDA: U.S. Food and Drug Administration
ICU: Intensive care unit
IDEAL: Innovation, Development, Evaluation, And Long-term implementation and monitoring
IND: Investigational new drug
IREB: Institutional Research Ethics Board
RCT: Randomized controlled trial
